# The complete mitochondrial genome sequence of Zhuanghe Big-boned chicken (*Gallus gallus*)

**DOI:** 10.1080/23802359.2020.1785351

**Published:** 2020-07-02

**Authors:** Jingjing Gu, Sheng Li

**Affiliations:** a College of Animal Science and Technology, Hunan Agricultural University, Changsha, China; b Hunan Provincial Key Laboratory for Genetic Improvement of Domestic Animal, Changsha, China; c Hunan Engineering Research Center of Poultry Production Safety, Changsha, China; d Maxun Biotechnology Institute, Changsha, China

**Keywords:** Zhuanghe Big-boned chicken, mitogenome, next generation sequencing

## Abstract

In this study, the complete mitochondrial genome of Zhuanghe Big-boned chicken (*Gallus gallus*) was obtained by using next generation sequencing method. The complete mitogenome sequence is 16,784 bp in length, containing 13 protein-coding genes, 2 ribosomal RNAs, 22 transfer RNA genes, and one control region. This work provides a valuable source of data for the study of the evolution of *Gallus gallus* mitochondrial genome.

Zhuanghe Big-boned chicken is a native breed in Liaoning Province of China. This chicken has the characteristics of big body size, strong foraging ability and large egg production. In 2000, Zhuanghe Big-boned chicken was determined as a national protected breed by the Ministry of agriculture of China. To investigate the genetic resource of this breed, we reported the complete sequence of the mitochondrial genome of Zhuanghe Big-boned chicken for the first time. The chicken sample used in this study was obtained in Zhuanghe City (39.68 N and 122.96 E), Liaoning Province, China. The total genomic DNA was extracted from the muscle specimen (Voucher No. DG150418) which stored at −80 °C in the Hunan provincial key laboratory for genetic improvement of domestic animal, Changsha, China. Sequencing libraries were generated from genomic DNA and sequenced on Illumina Hiseq 2500 platform. We generated a total of 12.52 Gb raw data and this sequence has been deposited in the NCBI Sequence Read Archive (SRA) with accession number SRR4292973. The complete mitochondrial genome was sequenced at 7790 times coverage. The assembled mitochondrial genome sequence was deposited in GenBank with accession number MT555046.

We analyzed the mitochondrial genome sequence of Zhuanghe Big-boned chicken using the total length of 16,784 bp. The mitogenome was annotated by tRNAscan-SE 2.0 (Chan and Lowe 2019) and MITOS (Bernt et al. 2013). The overall base composition of the complete mitogenome was 30.25% A, 23.72% T, 32.51% C, and 13.52% G and biased toward A + T nucleotides (53.97%). It contains the typical circular molecule structure, including 13 protein-coding genes (PCGs), 2 ribosomal RNA genes (rRNAs), 22 transfer RNA genes (tRNAs), and 1 noncoding control region (D-loop region). One protein-coding gene (ND6) and 8 tRNA genes were encoded on the light (L) strand. However, the other 12 protein-coding genes, 14 tRNA and 2 rRNA genes were encoded on the heavy (H) strand. The initiation codon of PCGs was ATG except for COX1 being GTG. There are four types of termination codon for PCGs, including TAA, TAG, AGG, and an incomplete termination codon “T”, which is the 5′ terminal of adjacent gene (Anderson et al. 1981). The longest protein-coding gene was ND5, which is 1818 bp, and the shortest was the ATP8, which is 165 bp. The 22 tRNA genes were distributed among rRNA and PCGs, ranging from 66 to 75 bp in length. The lengths of 12S rRNA and 16S rRNA genes were 977 bp and 1600 bp, respectively. In this study, the non-coding control region of the Zhuanghe big bone chicken mitogenome is 1231 bp long, accounting for 7.3% of the total length.

The neighbor-joining (NJ) phylogenetic tree was constructed based on the complete mitogenomes of 38 chicken breeds using Mega 7.0 (Kumar et al. 2016) with 1000 bootstrap replicates in order to infer the phylogenetic position of Zhuanghe Big-boned chicken. The results (Figure 1) shown that Zhuanghe Big-boned chicken has the closest relationship with the Jianmenguan Gray and grouped to one clade with Xuefeng, Huaiyang, Nandan and Guangxi Partridge. However, Zhuanghe Big-boned chicken is the longest distance with the Lindian. This work provides a valuable source of data for the study of the evolution of *Gallus gallus* mitochondrial genome.

**Figure 1.**
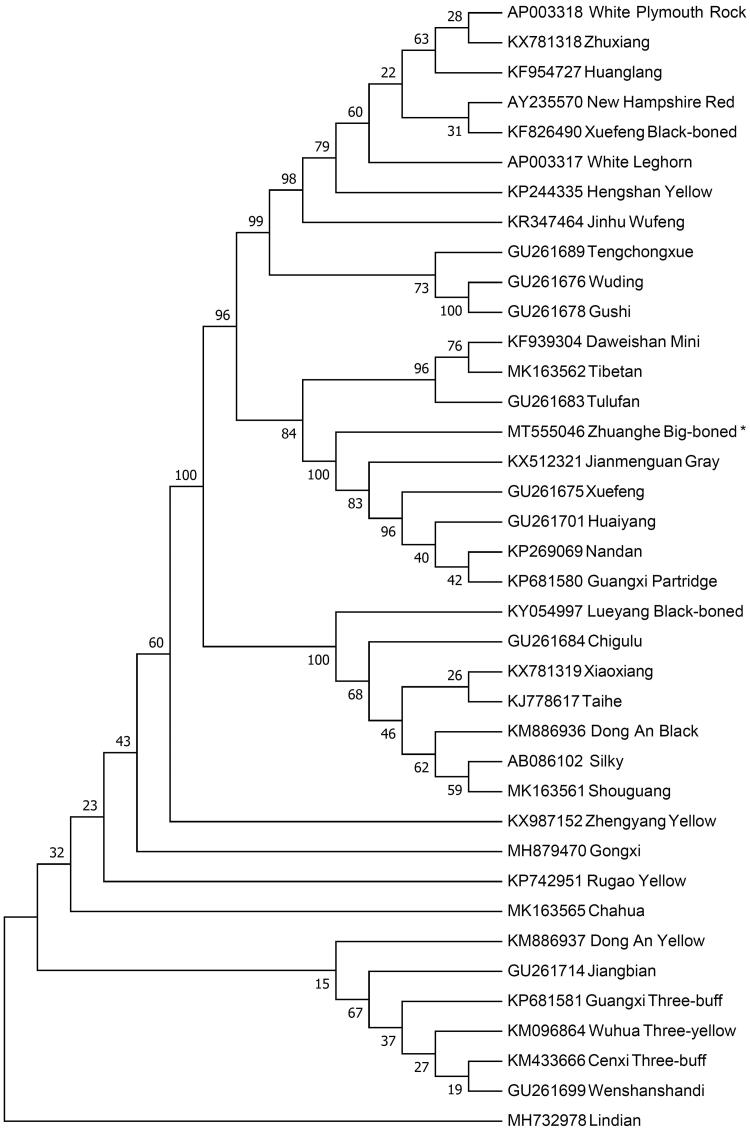
Neighbor-joining tree based on the complete mitochondrial DNA sequence of 38 chicken breeds. GenBank accession numbers are given before the species name.

## Data Availability

The sequence data that support the findings of this study are openly available in the NCBI Sequence Read Archive (SRA) at http://www.ncbi.nlm.nih.gov/sra/ with accession number SRR4292973. The complete mitochondrial genome of Zhuanghe Big-boned chicken (*Gallus gallus*) is openly available in GenBank at http://www.ncbi.nlm.nih.gov/genbank with accession number MT555046.
